# Insights Into the Inside – A Quantitative Histological Study of the Explosively Moving Style in Marantaceae

**DOI:** 10.3389/fpls.2018.01695

**Published:** 2018-12-05

**Authors:** Markus Jerominek, Maria Will, Regine Claßen-Bockhoff

**Affiliations:** ^1^School of Medicine and Health Sciences, Carl von Ossietzky University of Oldenburg, Oldenburg, Germany; ^2^Institute of Organismic and Molecular Evolution, Johannes Gutenberg University Mainz, Mainz, Germany; ^3^Institute of Biology and Environmental Sciences, Carl von Ossietzky University of Oldenburg, Oldenburg, Germany

**Keywords:** Marantaceae, movement, tensile stress, turgor, elastic energy, anatomy, cell volume reconstruction

## Abstract

This study aims to identify the histological basis for the extraordinary, fast movement of the style in Marantaceae. Although this explosive pollination mechanism was subject of many studies, quantitative measurements to document volumetric changes have never been conducted. Based on physical parameters and limitations (poroelastic time), the movement itself is by far too fast to be explained by turgor changes solely. Therefore, we address the hypothesis that the style contains elastic structures to store energy allowing the fast movement. We provide an experimental approach in *Goeppertia bachemiana* to identify histological differences of styles in various states, i.e., steady, unreleased, and released state. Cross and longitudinal sections were used to reconstruct the cell volume in different sectors of the style. Histological data were discussed with respect to a putative water shift (turgor movement) and elastic instabilities that were proposed to explain the style movement of Marantaceae. Current data show, that the upper epidermis is under tensile stress in the unreleased state. After style release, the lower side of the style revealed an enormous water up-take. According to our results, we hypothesize that the fast style movement of *G. bachemiana* is likely based on an elastically stretched upper epidermis, whereas a “soaking tissue” at the lower side presumably mediates the up curling of the style. The experimental data show that at least for *G. bachemiana*, physical limitations such as the poroelastic time are suitable parameters to predict movements that are based on elastic instabilities.

## Introduction

Although fast movements are largely uncommon to occur in plants, the latter have various complex mechanisms to allow for a movement of organs (Bünning, 1959). One well-known example is the carnivorous Venus flytrap (*Dionaea muscipula* J.Ellis), a model system for turgor movements (Haupt, 1977). While the movement of fly catching leaves are known for centuries (Darwin, 1875) the underlying mechanism was reconsidered later and is referred to as “snapping” (Hodick and Sievers, 1989) or more recently as “snap-buckling movement” (Forterre et al., 2005).

**FIGURE 1:**
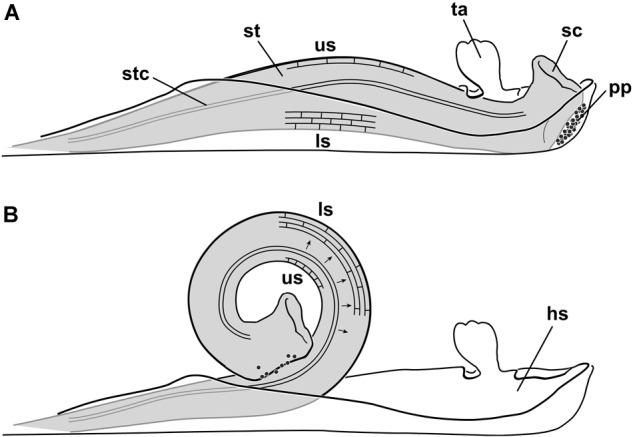
Explosive style mechanism and histology of the cells on the upper (us) and lower side (ls) of the style. **(A)** Unreleased state, style (st) growing against the hooded staminode setting up compressive stress in the lower cells; cells at the upper side expanded; **(B)** released state, after deflecting the trigger appendage the style curls up; lower cells are expanded and the upper ones are shortened in length. Black arrows indicate a water shift from cells of the upper to the lower side (Pischtschan and Claßen-Bockhoff, 2010); hs, hooded staminode; ls, lower side; pp, pollen plate; sc, stigmatic cavity; st, style; stc, style channel; ta, trigger appendage; us, upper side (modified from Pischtschan and Claßen-Bockhoff, 2010).

Another example of a unique movement in plants is the explosive pollination mechanism of Marantaceae (Kennedy, 1999). In this tropical plant family, the style is enveloped and kept under tension by a hooded staminode (Figure 1, hs) (compressive stress). Based on the deflection of a trigger appendage (ta), the hooded staminode can be removed by pollinators and the stored tension is relieved. As a consequence, the style irreversibly curls up in a split second and pollen (pp) is transmitted to the insect’s body (Claßen-Bockhoff, 1991). It took 50 years of investigation to understand the pollination mechanism (Lindley, 1819, 1826; Nees von Esenbeck, 1831; Gris, 1859; Delpino, 1869) but the biomechanics on which the style bending is based on, is still unknown.

Recent histological studies showed that the style of Marantaceae contains an upper layer with elongated cells (us) and a lower one (ls) with cells that are under pressure (Figure 1A; Claßen-Bockhoff and Pischtschan, 2000; Pischtschan and Claßen-Bockhoff, 2010). Since the length of the upper cells decrease and the lower cells increase in length after style movement (Figure 1B), Pischtschan and Claßen-Bockhoff (2010) assumed a water shift through the style to account for the observed style bending. The mechanism could thus be comparable to a common turgor movement as described for *Aldrovanda vesiculosa* L. (Droseraceae) (Ashida, 1934 but see also Westermeier et al., 2018), *Biophytum sensitivum* (L.) DC. (Oxalidaceae) (Umrath, 1928), *Desmodium gyrans* (L.f.) DC. (Fabaceae) (Antkowiak et al., 1992), *Mimosa pudica* L. (Fabaceae) (Weintraub, 1952), or *Sparrmannia africana* L.f. (Tiliaceae) (Bünning, 1934).

This putative “hydraulic mechanism” (Pischtschan and Claßen-Bockhoff, 2010) was recently tested in physical, chemical, mechanical, and electrophysiological experiments (Jerominek and Claßen-Bockhoff, 2015). Based on these studies, it is evident that the mechanism releases the style mechanically. To test the hypothesis on a water shift inside the style, it is necessary to reconstruct the cell volume before and after the movement. Today, methods such as confocal microscopy (Gray et al., 1999; Franks et al., 2001; Fernandez et al., 2010) or μ-computer tomography (Forsberg et al., 2008; Staedler et al., 2013) offer new possibilities for cellular measurements. However, confocal microscopy is not suitable for our study due to the high reflections on the epidermis and μ-computer tomography is not yet precise enough for studies at cell level.

From a theoretical point of view, the explosive style movement is much too fast to be mediated solely by a turgor based water shift (Skotheim and Mahadevan, 2005). Pischtschan and Claßen-Bockhoff (2010) argue that according to Skotheim and Mahadevan (2005) the movement must be based on elastic instabilities, e.g., “snap-buckling.” For such movements it is possible to use the poroelastic time (τ_p_; Eq. 1) as approximate value. This parameter describes the physical limit of diffusive equilibration of pressure via fluid transport depending on the size of the moving tissue (L = smallest dimension), the elastic modulus (E), the viscosity (μ) and the hydraulic permeability (k) according to Skotheim and Mahadevan (2005).

(1) $$\begin{array}{c} \tau_{p}{\sim \mu L}^{2}/kE \end{array}$$

According to Eq. 1 and the average style diameter (L = 1 mm; estimation based on own morphometric measurements), the poroelastic time for *Goeppertia* is 1.6 s (τ_p_/L^2^ = 1.6 s/mm^2^ a typical value for soft plant tissue, see Skotheim and Mahadevan, 2005). Indeed, the duration of style movement of Marantaceae is only 0.2–0.03 s (Kunze, 1984; Claßen-Bockhoff, 1991) and is thus shorter than the calculated poroelastic time of this tissue. Consequently, a solely turgor based explanation has to be rejected. In fact, elastic instabilities must be involved in this particular movement of plant tissue.

To test this hypothesis, it is necessary to quantify changes in cell size (volume) and shape before and after the style is released. The current approach addresses a turgor-based movement (hydraulic mechanism) via histological investigations. Additionally, structures have to be identified that might account for a storage of elastic energy, e.g., vascular bundles (VBs) or cell wall reinforcement.

In the current study, we used a conventional histological approach to measure cell dimensions based on the “Duchartre-Lewis method” (Flint and Matzke, 1948). The aim is to understand the histological basis for the style movement and to identify putative structures that might store elastic energy.

## Materials and Methods

### Plant Material

**FIGURE 2:**
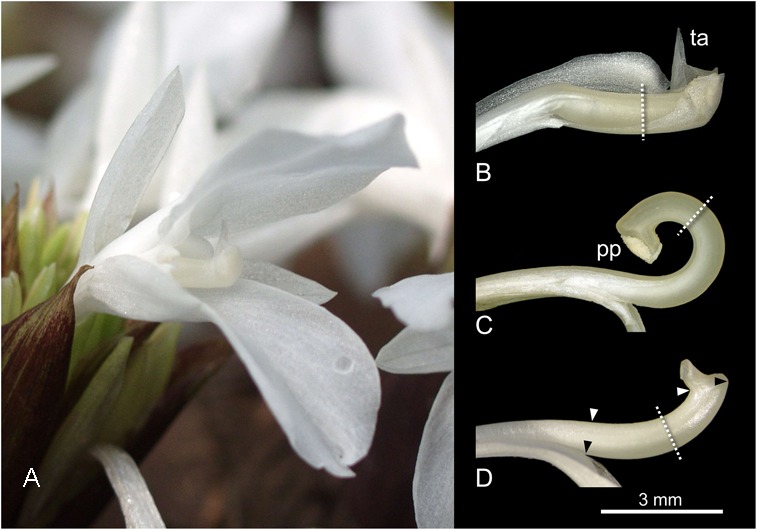
Flower **(A)** and dissected styles **(B–D)** of *Goeppertia bachemiana*. **(B)** Unreleased style under tension, enveloped by the hooded staminode. **(C)** Released state; style is curled up. **(D)** Steady state manipulation; style only slightly curved. Dotted lines indicate orientation of the cross sections within the bending zone. Triangles indicate the boundaries for the length measurements on the lower (black triangles, from fusion zone to the knob of the style head) and upper side (white triangles, from a point perpendicular to the fusion zone to the beginning of the style head). ta, trigger appendage; pp, pollen plate.

For histological investigations flower material of *Goeppertia bachemiana* (E. Morren) Borchs. & S. Suárez (Figure 2A; syn. *Calathea bachemiana* E. Morren) was collected in the greenhouse of the Botanical Garden of the Johannes Gutenberg University (Mainz, Germany). For plant identification the Flora Brasiliensis (Petersen, 1890) was used. The species grows clonally. Thus, all flowers are genetically identical to a single “mother” plant.

### Preparation of Samples

To reconstruct the assumed shift of water during style movement three different states were analyzed, i.e., unreleased (Figure 2B), released (Figure 2C), and steady state (Figure 2D). To get the latter, the hooded staminode was removed in bud stage 1 day before flowering according to Pischtschan and Claßen-Bockhoff (2008). At this time, tension is not yet set up and the style grows without any external pressure.

To minimize shrinking artifacts (Talbot and White, 2013), flowers of all states were collected shortly after anthesis in the early morning and fixed in 50% formalin-acetic acid alcohol (FAA). The latter was preferred over other chemical solutions that lead to tissue shrinking (ethanol) (Talbot and White, 2013) or an increased fragility of the tissue (methanol) (Pischtschan, 2007). Furthermore, alcohol often leads to style release during fixation already described for some Marantaceae species (Claßen-Bockhoff and Heller, 2008; Pischtschan and Claßen-Bockhoff, 2010).

After a minimum of 2 weeks to allow for a complete fixation, i.e., a substitution of water inside the cells against FAA, styles were isolated from the flowers. For embedding, the plant tissue was dehydrated in absolute ethanol, transferred to isobutyl alcohol (IBA) and infiltrated in paraffin. Cross and longitudinal sections (thickness: 15 μm) were made with a rotary microtome (Leitz, Germany) and affixed on glass slides. Fully dried slides were stained in 0.05% toluidine blue for 10 min (O’Brien et al., 1964) and washed with distilled water. Afterward, paraffin was removed with Roti^®^-Histol (ROTH, Germany) and slides were sealed with Eukitt^®^ (ROTH, Germany).

### Graphical Analysis

**FIGURE 3:**
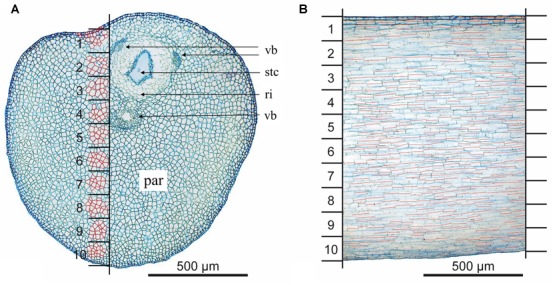
Determination of the sectors to reconstruct the cell area and length. **(A)** Cross section of the style; 10 sectors are indicated by black horizontal lines; measurement of cell areas is exemplarily shown (red highlighted cells); **(B)** longitudinal section; cell length is indicated by red lines; par, parenchyma; ri, ring of cells surrounding the style channel; stc, style channel; vb, vascular bundle.

To reconstruct changes in cell volume, the area (cross section) and the length (longitudinal section) of individual cells from the same sector (Figure 3) were analyzed. Cross sections were made in the bending zone (Figures 2B–D, dotted lines) for eight unreleased, eight released and four styles in steady state. Cross section areas of single cells were measured for ten different sectors defined by a mask (Figure 3A). The latter was adjusted using the VBs (Figure 3A; vb). For each sector, an average of 15 cell areas was analyzed. Upper epidermal and sub-epidermal cells were measured separately. Lower epidermal cells were excluded from further calculation since they were that much stretched in the released state that a proper measurement was no longer possible. For analyses, cell walls were retraced in Illustrator CS3 (Adobe). The enclosed cell areas (*N* = 3247) in the cross section were measured with Photoshop CS3 (Adobe) and the according number of pixels were converted in μm^2^.

To measure the corresponding cell length within these 10 sectors, vertical longitudinal sections in the area of the mask were analyzed with Imagic (ims Client 12v). Measurements were performed only for well-noticeable cells (*N* = 4057, Figure 3B) in four styles per state.

### Correction of Data

Artificial shrinking of the style tissue due to fixation (Talbot and White, 2013) mainly affected the length of the style. To correct the corresponding data of cell length, 10 fixed and 10 untreated styles were compared for each state. Thereby, the length of the upper and lower side of the styles were measured (Illustrator CS3, Telegraphics path lengths Plugin). The lower length is defined from the fusion zone of the hooded staminode and style to the knob of the style head (black triangles, Figure 2D). The upper style length was measured from a point perpendicular to the fusion zone to the beginning of the style head (white triangles, Figure 2D). To reconstruct the length of the ten sectors the difference between the upper and lower length was equally split and assigned to the sectors (Supplementary Table S2). Thereby the relative position (Rel. Pos.) of the sector was multiplied with the difference of upper and lower length and added to the length of the upper side (Difference × Rel. Pos. + Length upper side). Afterward, for each sector the length of untreated styles was divided by the length of fixed ones to obtain the corresponding correction factor.

### Reconstruction of Cell Volume

To reconstruct the cell volume, the cell length and area were multiplied for each state and sector. Longitudinal and cross sections were analyzed and the volume of cells in different layers of the style was reconstructed according to Pütz et al. (1990). Here, we used the medians instead of the mean value to minimize the effect of outliers. Relative changes of the released and steady state to the unreleased state were calculated as percentage.

### Vascular Bundles

**FIGURE 4:**
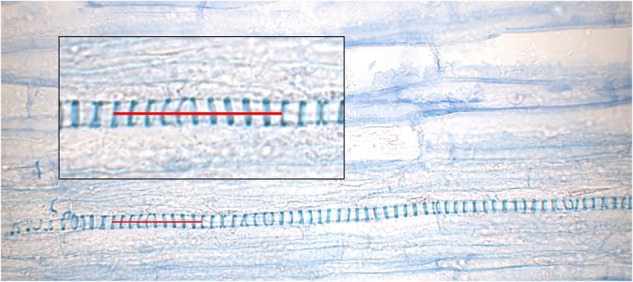
Longitudinal section trough an unreleased style. A close-up of a vessel from the upper vascular bundle embedded in the parenchyma is provided in the box; a red line indicates 10 helical loops.

As a potential source for elastic energy, the VBs were analyzed by their length. To compare all three states, 637 individual vessels from four individual styles per state were recorded. For each vessel, the length of 10 helical loops (Figure 4) was digitally measured using Imagic (ims Client 12v).

### Statistical Analysis

Significant changes of the cell length and area were tested with a two-sample *t*-test. As a precondition for the *t*-test, data were tested for a normal distribution with the Kolmogorov–Smirnov test (K–S test). For data that was not normally distributed a Mann–Whitney *U* test was used instead of a *t*-test. All statistics were performed with SPSS ver. 20.0.0.1.

## Results

### Style Length Differences Indicate Tissue Shrinking After Fixation

**FIGURE 5:**
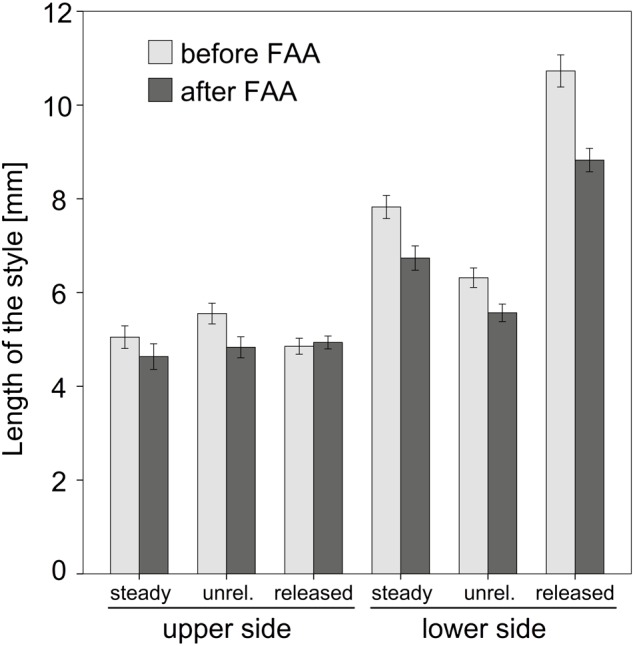
Comparison of the style length before and after FAA-fixation. Mean values and standard errors for steady, unreleased (unrel.), and released state are provided for upper and lower side of the style. Except for the upper side of the released style FAA led to a shrinking of the tissue. Bars represent means ± standard error (see Supplementary Table S1).

Length measurements of the upper and lower side of the style provided useful data to interpret the explosive style movement. A comparison of the length of unfixed styles clearly showed that the upper side significantly decreased in length (T_18_ = 5,684; *P* = 0,000) while the lower side increased in length (T_18_ = -24,924; *P* = 0,000) during style movement (Figure 5). Values for the steady state samples were intermediate, i.e., between the length of the released and unreleased state (Supplementary Table S1). Measurements after FAA-fixation showed the same proportion as non-fixed styles for the lower side. In contrast, the upper side was rather constant in all three states after fixation (Figure 5).

The comparison between fixed and non-fixed styles showed a significant tissue shrinking due to dehydration, except for the upper side of released states (Figure 5 and Supplementary Table S1). The corresponding factors to correct these particular length differences related to fixation were up to 1.21 (e.g., released state measurements for sector 10; Supplementary Table S2).

### Histology of the Style as Basis for Cell Volume Reconstruction

**FIGURE 6:**
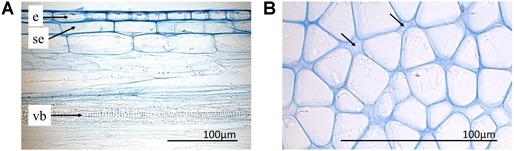
Histological sections with toluidine blue staining. **(A)** Upper side of the style (longitudinal section) with epidermis (e), sub-epidermis (se), and one vessel (ve) of the upper vascular bundle. **(B)** Parenchyma of the lower part of an unreleased style in the bending zone (cross section). Intercellular spaces indicated by arrows.

Epidermal cells were only investigated in the upper side of the style since the ones on the lower side were irreversibly stretched after style release. In general, epidermal cells have a cylindrical shape and were thick-walled (e; Figure 6A). The three VBs (vb) are arranged around the style canal in the upper part of the style (Figure 3A), a larger one below and two smaller ones above or lateral to the style channel (stc), respectively. The style channel is characterized by a layer of thick-walled glandular cells, which were intensively colored after staining. These cells are surrounded by a ring-shaped tissue (ri) with cells of low light density and thus pale blue colored after staining with toluidine blue (Figure 3A). Together, style channel and VBs are embedded in parenchyma cells (par) which are highly turgescent and have an elongated cylindrical shape in the longitudinal section (Figure 3B). Intercellular spaces are observed between the parenchyma cells (arrows; Figure 6B).

### Cell Dimensions

Since the anticlinal cell walls of all cell layers (i.e., E, SE and sectors 1–10) are parallel to each other (Figure 6A) it is possible to calculate the volume based on a straight prism. A given change in cell length does not necessarily lead to a change of the cell volume, as it will be shown for the epidermal and sub-epidermal layers.

#### Epidermal (E) and Sub-Epidermal (SE) Layers

**FIGURE 7:**
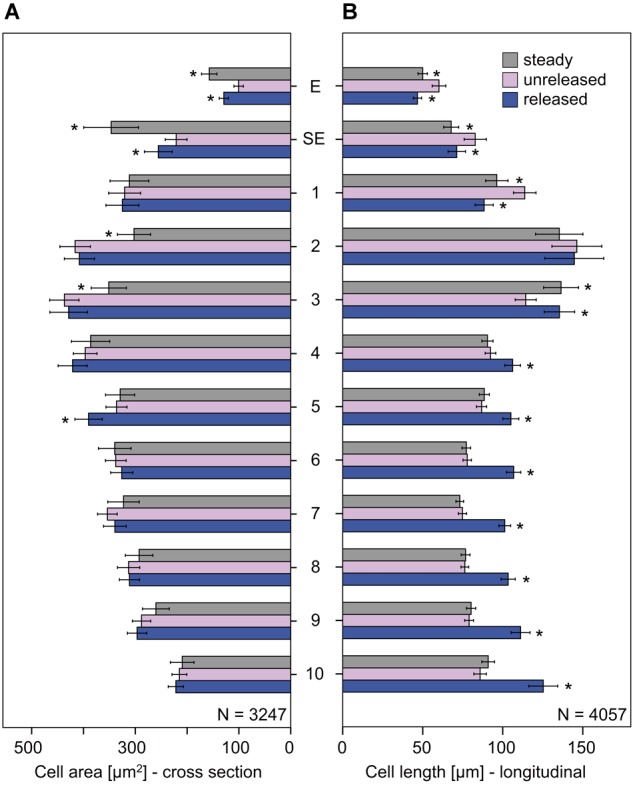
Cellular measurements of the style of *G. bachemiana*: **(A)** Cell area (in μm^2^) and **(B)** cell length (in μm). Mean values and standard errors for steady state, unreleased, and released state are provided for epidermal (E) and sub-epidermal cells (SE) and sectors 1–10. An asterisk indicates significant differences (in comparison to the unreleased state) of the steady and released state. Bars represent means ± standard error (see Supplementary Tables S3, S4).

**FIGURE 8:**
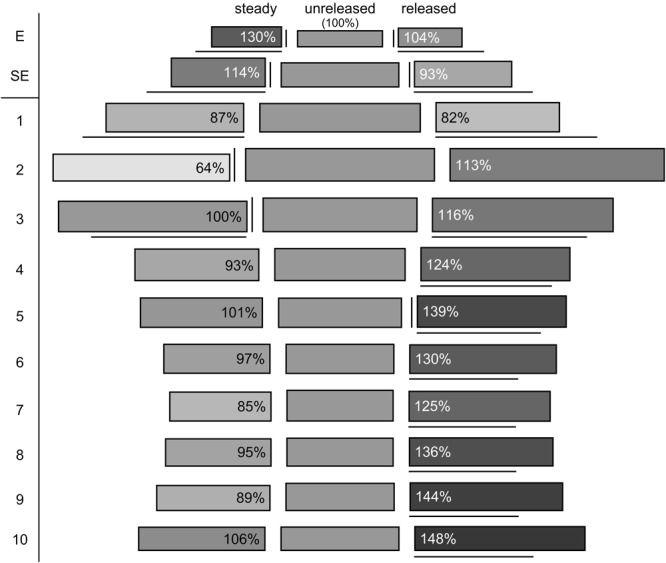
Reconstruction of cell volumes (gray shaded boxes). Cell area is represented by height and cell length by length of the boxes. The corresponding cell volume is illustrated by the rectangular and referred to the unreleased state (middle; volume corresponds to 100%), relative changes are indicated by different shades of gray and the percentages provided in the box. Significant differences compared to the unreleased state are indicated by vertical (cell area) and horizontal lines (cell length). E, epidermis; SE, sub-epidermis.

Cells in the unreleased state clearly differ from those of the steady and released state, i.e., by their cell area and length (Figures 7, 8 and Supplementary Tables S3, S4). While the areas of the epidermal cells significantly increased in the released and steady state (Figure 7A), their length (Figure 7B) decreased. The shape of cells in the steady and released state are more similar to each other than to cells in the unreleased state.

The observed changes lead to a deformation in the two layers from rather elongated to shortened prismatic cells (Figure 8). As cell area and length change, it is possible that cells remain the same volume while changing their forms. This was shown for epidermal cells (E) in the released versus unreleased state (Figure 8). However, comparing steady and unreleased states for E and SE, the change of the cell forms indeed goes along with a difference of the volume (i.e., 14% in the SE and 30% in the E; Figure 8).

#### Sectors 1–10

In the cells below the epidermal and sub-epidermal layers, i.e., sectors 1–10, the cell areas were of comparable size in all three states (Figure 8). Significantly different cell areas, indicated by vertical lines in Figure 8, and by asterisks in Figure 7, respectively, were only observed for sector 2, 3, and 5 (Figures 7, 8 and Supplementary Table S3).

As to the cell length, sectors 1–3 showed a reverse elongation. In comparison to the unreleased state, the steady and released states decreased in sector 1 but increased in sector 3. Sector 2, which has the longest cells all over the style, showed no significant change in cell length between all the three states (Figures 7, 8 and Supplementary Table S4).

Sectors 4 to 10 were significantly longer in the released state than in the unreleased state but showed no significant change in the steady state (Figures 7, 8 and Supplementary Table S4). Since the cell area remained constant, the corresponding cell volume in the released state was higher (Figure 8).

The corresponding cell volume (Figure 8) increased in the lower part of the style (sector 2–10: 72709 μm^3^; Supplementary Table S5) comparing the unreleased and released state. In contrast, the upper part decreased (sector 1: -6139 μm^3^; Supplementary Table S5). Volumetric changes between the steady and unreleased states are rather little in the lower part of the style. Only in sector 1 and 2 the steady state has a decreased volume (sector 1: -4733 μm^3^; sector 2: -18716 μm^3^; Supplementary Table S5).

### Vascular Bundles (VBs)

**FIGURE 9:**
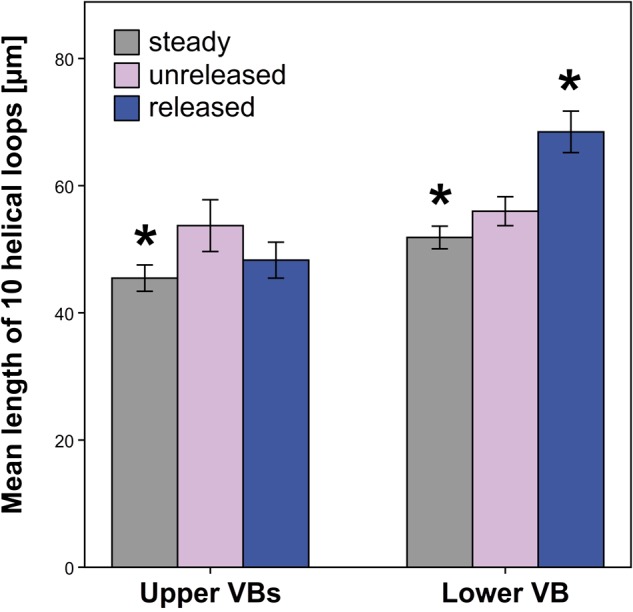
Length of vascular bundles (VBs) in *G. bachemiana* for all three states. Significant differences (*P* ≤ 0.05, *U*-test) compared to the unreleased state are indicated by an asterisk. Bars represent means ± standard error (see Supplementary Table S6).

Significant length differences in the VBs were observed between the unreleased and released state only for the lower VB (Figure 9 and Supplementary Table S6). The latter increased in length after style release. In contrast, the upper VBs decreased in length when the style is released but this change is not significant. Styles, which grew without the external tension of the hooded staminode (steady state), had upper and lower VBs significantly shorter than in the unreleased state (Figure 9 and Supplementary Table S6).

## Discussion

Current data contribute to a deeper understanding of the post-stimulation processes resulting in the explosive style movement in Marantaceae. The fast style movement is based on a tensile stress of the upper epidermis and a particular tissue at the lower side of the style. We refer to this tissue as “soaking tissue” since it is able to absorb water in a very short time. The corresponding cells increase in length and thus in their volumes. A water shift from the upper to the lower side of the style can only partly explain the massive volumetric changes observed.

### Vascular Bundles – Compressed or Stretched?

Niklas (1992) discussed VBs to operate mechanically like springs in, e.g., *Narcissus tazetta* L. (Amaryllidaceae) and *Cyclamen hederifolium* Aiton (Primulaceae). Based on this model, it is likely that VBs are also involved in the storage of elastic energy in Marantaceae. A comparison of the length of VBs among the released, unreleased, and steady state (Figure 9) should clarify how far the style movement and corresponding tissue deformation are based on an elastic tension stored in the VBs. While a tensile stress could be identified by a reduced length of vessels in the relaxed states (steady and released), a compressive stress would be indicated by an elongation of VBs after release.

Since the upper VBs decrease in length in both relaxed states (Figure 9), one can assume a tensile stress here. However, the lower VB shows conflicting results for the steady (shorter VB) and released state (elongated VB). This incongruence indicates that the lower VB is neither elastically stretched nor compressed. Assuming that the VBs act in the same way, we conclude that the style movement passively deforms all of them.

Current histological analyses support this hypothesis. They reveal that the secondary walls of the tracheary elements are ring-like in *G. bachemiana* (Figure 4) instead of showing helical (spiral) thickenings like springs as already described by Evert and Eichhorn (2006). Consequently, the mechanical properties as proposed by Niklas (1992) for spring-like VBs can be rejected for *G. bachemiana*. The VBs are rather passively elongated (lower VB) or shortened (upper VBs) when the style curls-up. The significantly shorter vessels in the steady state (relaxed) clearly show that upper and lower VBs are passively elongated in the unreleased state. This elongation might be due to the stretched upper side of the style.

### Elastic Energy Stored in the Upper Epidermal Cells Speeds Up the Style

Measurements for the upper epidermal and sub-epidermal layers clearly show that the cell shape changes dramatically after style release. Thereby, the cell length is decreasing while the cell area is increasing. However, the cell volume remains constant. This finding indicates that there is no water in- or out-flux (shift) in the cells and the deformation is thus not turgor-based. Due to the incompressible cell fluid, external strains can only lead to a deformation but not to a volumetric change (Nilsson et al., 1958). This is in accordance with experiments performed by Lockhart (1967) who addressed this aspect in cells of mung bean seedlings. Referring these results to Marantaceae, the different shapes of the epidermal cells before and after style movement is either based on (1) a tensile stress in the unreleased state or (2) on a compressive stress in the released state due to the elongation of the lower side.

Based on histology, cell walls of the style of *G. bachemiana* differ. The epidermal cell walls are thicker than those in the parenchyma (Figure 6) and might allow for the elastic strain and storage of energy. Similar observations are known from *Impatiens parviflora* DC. (Balsaminaceae) (Overbeck, 1924). Here, the inner layer of its exploding fruits has thickened cell walls storing strain energy. We assume that in Marantaceae the epidermal cells are elastically stretched and thus under tensile stress in the unreleased state.

Evidence for the current assumption is provided by the comparison of fixed and unfixed styles. The application of FAA reduces the turgor in the whole style (reduced style length) until a movement is not possible anymore. In this condition, any tensile or compressive stress is reduced or even completely eliminated in the style. Since the length of the upper side of the style shows no significant change after FAA fixation in the released styles (Figure 5), this state seems to be not affected by compressive stress. In contrast, the upper side of unreleased state decreases after fixation and clearly indicates a tensile stress.

Although this aspect was not in the scope of the present study, it is obvious that one should attach importance to the cell wall. To confirm a tensile stress in the epidermal layers, sub-microscopic investigation of the cell wall might be useful to distinguish different elastic states (Frey-Wyssling, 1936; Fleurat-Lessard, 1990; Suslov and Verbelen, 2006). Hodick and Sievers (1989) investigated cross sections of the Venus flytrap in a polarizing microscope and demonstrated that the microfibril orientation seems to be important for the movement and the corresponding stretching of the cells. Indeed, it was the cell walls of the upper and the lower epidermis as well as the mesophyll layer under the upper epidermis showing a preferential microfibril orientation in the direction of the applied stress. Compared to our data, the same layers (E, SE, and section 1) seem to be involved here. Furthermore, the composition of the cell wall (Cosgrove, 2005, 2014) as well as chemical or enzymatical processes might be more complex in cells exposed to mechanical strains as addressed (Sapala et al., 2018).

Cutting experiments as conducted by Hodick and Sievers (1989) or Dumais and Steele (2000) might provide clear evidence for tissue stresses. Experiments addressing the sensitivity of the style of Marantaceae (unpublished data) give a first insight. When the upper side of the style was cut transversely, the upper layers (at least E and SE) curl up and separate from the lower layers. These preliminary findings further support the assumption of a tensile stress in the epidermis.

Furthermore, the cellular measurements of the steady state can be used. However, it is important to consider, that the growing conditions differ in this state, i.e., growing without hooded staminode and thus without external strains. It is evident that the epidermal cells in the steady and the released state resemble each other (Figure 8). Both represent relaxed states and have clearly shorter epidermal cells than the unreleased state (Figure 8). Therefore, it has to be assumed that the cells in the unreleased state are stretched. Consequently, the latter are under tensile stress. So far, it remains unclear why the upper side of the steady state decreases after FAA-fixation. We assume that this change in length is most likely based on the turgor decrease during fixation. Additionally, the epidermal cells show higher volumes in the steady state than the unreleased state (Figure 8). The missing tension of the hooded staminode obviously allows for an increased cell elongation and volume. Nevertheless, it is interesting to further investigate why the unreleased state has smaller cells especially since this finding is in conflict with Cleland (1971). He stated that cell walls extend when affected by a constant force. In Marantaceae, this force might be comparable to the one, mediated by the hooded staminode in the unreleased state.

Our findings show that the upper epidermal cells are most likely under tensile stress in the unreleased state. As soon the hooded staminode is removed, the elastic energy is released and the style curls up (Figure 1).

### A “Soaking Tissue” Mediates the Curl-Up of the Style

In contrast to the upper side of the style, cells below the style channel (sectors 4–10) increase in volume up to 148% when the style is released (Figure 8). This is based on a shift of water as proposed by Pischtschan and Claßen-Bockhoff (2010) but current data reveal that although cells of sector 1 show a significant water loss (-6139 μm^3^; Supplementary Table S5), the latter is by far too low to explain the observed increase of cell volumes on the lower side of the style (72709 μm^3^; Supplementary Table S5). Kunze (1984) assumed that water might be transported within the style. He suggested that it rather comes from the upper side of the style and is transported into the intercellular space. However, the water loss in sector 1 alone is not suitable to explain the movement as our data indicate. Although, we confirm that water is responsible for the increase of cell volumes in the lower side of the style. Further experiments are necessary to test whether the water might come from the intercellular space and the cell walls, respectively.

One hint for such a water shift is the model of tissue construction by Pischtschan and Claßen-Bockhoff (2010). The authors described “strongly perforated cell bundles” that might support the stability (collenchyma), pliability (intercellular spaces), and water transport (extremely porous tissue) in several species of Marantaceae. In *G. bachemiana*, another character might contribute to increase the pliability of the style in the investigated species, i.e., cells in sector 2 show no significant change in their length. They are located between the two functional tissues, the decreasing upper layers (E, SE, and sector 1) and the increasing lower layers (sector 3–10). Consequently, the cells in sector 2 are not able to change the cell shape longitudinally (cell length) but vertically (cell area). This might explain the low volume in the steady state that is based on an increased cell area in sector 2.

So far, we conclude that the cells of the lower layers, i.e., sector 3–10, are capable to absorb water. We refer to these cells as a “soaking tissue” in the lower side of the style, which is involved in a rapid and massive water shift that leads to an irreversible curl-up of the style.

## Conclusion

The quantitative histological analysis reveals that the style movement is based on two components, the elastically stretched upper epidermis and a particular tissue in the lower side of the style (“soaking tissue”). The elastic instabilities in the epidermis allow for the fast movement and the “soaking tissue” supports the curling of the style. Since the volumetric changes in the lower layers are higher than the water loss in the upper layers, we assume that also water from the apoplast might be involved.

Experimental data clearly show that at least for *G. bachemiana*, physical limitations such as the poroelastic time are suitable parameters to predict fast movements based on elastic instabilities.

How far the same principle is realized in the whole family remains an open question. Based on the high phenotypical diversity of floral structures, it is possible that at least species with small flowers lack the elastic component and solely rely on the extension of the “soaking tissue.”

Beyond knowing the histological basis of this explosive mechanism, it might be useful for applied engineering or biomimetics, e.g., to design a material allowing for a movement without the need for joints.

## Author Contributions

MJ and RC-B initiated the study, designed the experimental setup, and analyzed and interpreted the data. MJ performed data acquisition, conducted the statistics, and drafted the manuscript. RC-B and MW revised the manuscript.

## Conflict of Interest Statement

The authors declare that the research was conducted in the absence of any commercial or financial relationships that could be construed as a potential conflict of interest.

## References

[B1] Antkowiak B. Engelmann W. Herbjørnsen R. Johnsson A. (1992). Effects of vanadate, N2 and light on the membrane potential of motor cells and the lateral leaflet movements of *Desmodium motorium*. *Physiol. Plant.* 86 551–558. 10.1111/j.1399-3054.1992.tb02169.x

[B2] Ashida J. (1934). Studies on the leaf movement of *Aldrovanda vesiculosa* L. *Protoplasma* 22:155 10.15281/jplantres1887.51.505

[B3] Bünning E. (1934). Elektrische Potentialänderungen an seismonastisch gereizten Staubfäden. *Planta* 22 251–268. 10.1007/BF01916323

[B4] Bünning E. (1959). “Physiology of movements,” in *Encyclopedia of Plant Physiology* ed. Ruhland W. (Berlin: Springer).

[B5] Claßen-Bockhoff R. (1991). Untersuchungen zur Konstruktion des Bestäubungsapparates von *Thalia geniculata* (Marantaceen). *Bot. Acta* 104 183–193. 10.1111/j.1438-8677.1991.tb00215.x

[B6] Claßen-Bockhoff R. Heller A. (2008). Style release experiments in four species of Marantaceae from the Golfo Dulce area, Costa Rica. *Stapfia* 88 557–571.

[B7] Claßen-Bockhoff R. Pischtschan E. (2000). “The explosive style in Marantaceae-preliminary results from anatomic studies,” in *Plant Biomechanics 2000* eds Spatz H. C. Speck T. (Stuttgart: Thieme) 515–521.

[B8] Cleland R. (1971). Cell wall extension. *Annu. Rev. Plant Physiol.* 22 197–222. 10.1146/annurev.pp.22.060171.001213

[B9] Cosgrove D. J. (2005). Growth of the plant cell wall. *Nat. Rev. Mol. Cell Biol.* 6 850–861. 10.1038/nrm1746 16261190

[B10] Cosgrove D. J. (2014). Re-constructing our models of cellulose and primary cell wall assembly. *Curr. Opin. Plant Biol.* 22 122–131. 10.1016/j.pbi.2014.11.001 25460077 PMC4293254

[B11] Darwin C. R. (1875). *Insectivorous Plants.* London: John Murray.

[B12] Delpino F. (1869). Breve cenno sulle relazioni biologiche e genealogiche delle Marantaceae. *Nuovo G. Bot. Ital.* 1 293–306.

[B13] Dumais J. Steele C. R. (2000). New evidence for the role of mechanical forces in the shoot apical meristem. *J. Plant Growth Regul.* 19 7–18. 10.1007/s003440000003 11010988

[B14] Evert R. F. Eichhorn S. E. (2006). *Esau’s Plant Anatomy: Meristems, Cells, and Tissues of the Plant Body: Their Structure, Function, and Development.* Hoboken, NJ: Wiley and Sons 10.1002/0470047380

[B15] Fernandez R. Das P. Mirabet V. Moscardi E. Traas J. Verdeil J.-L. (2010). Imaging plant growth in 4D: robust tissue reconstruction and lineaging at cell resolution. *Nat. Methods* 7 547–553. 10.1038/nmeth.1472 20543845

[B16] Fleurat-Lessard P. (1990). “Structure and ultrastructure of the pulvinus in nyctinastic legumes,” in *The Pulvinus: Motor Organ for Leaf Movement* eds Satter R. L. Gorton H. L. Vogelmann T. C. (Washington, DC: The American Society of Plant Physiologists) 101–129.

[B17] Flint T. J. Matzke E. B. (1948). Recent procedures for the study of three-dimensional shapes of resting cells, and a new method for the shape study of cells in division. *Science* 108 191–192. 10.1126/science.108.2799.191 17821153

[B18] Forsberg F. Mooser R. Arnold M. Hack E. Wyss P. (2008). 3D micro-scale deformations of wood in bending: synchrotron radiation μCT data analyzed with digital volume correlation. *J. Struct. Biol.* 164 255–262. 10.1016/j.jsb.2008.08.004 18804168

[B19] Forterre Y. Skotheim J. M. Dumais J. Mahadevan L. (2005). How the Venus flytrap snaps. *Nature* 433 421–425. 10.1038/nature03185 15674293

[B20] Franks P. J. Buckley T. N. Shope J. C. Mott K. A. (2001). Guard cell volume and pressure measured concurrently by confocal microscopy and the cell pressure probe. *Plant Physiol.* 125 1577–1584. 10.1104/pp.125.4.1577 11299339 PMC88815

[B21] Frey-Wyssling A. (1936). Über den optischen Nachweis der Turgorstreckung. *Ber. Dtsch. Bot. Ges.* 54 445–454. 10.1111/j.1438-8677.1936.tb01983.x

[B22] Gray J. D. Kolesik P. Høj P. B. Coombe B. G. (1999). Confocal measurement of the three-dimensional size and shape of plant parenchyma cells in a developing fruit tissue. *Plant J.* 19 229–236. 10.1046/j.1365-313X.1999.00512.x 10476070

[B23] Gris A. (1859). Observations sur la fleur des Marantées. *Ann. Sci. Nat. IV* 12 193–219.

[B24] Haupt W. (1977). *Bewegungsphysiologie der Pflanzen.* Stuttgart: Thieme.

[B25] Hodick D. Sievers A. (1989). On the mechanism of trap closure of venus flytrap (*Dionaea muscipula* Ellis). *Planta* 179 32–42. 10.1007/BF00395768 24201419

[B26] Jerominek M. Claßen-Bockhoff R. (2015). Electrical signals in prayer plants (Marantaceae)? Insights into the trigger mechanism of the explosive style movement. *PLoS One* 10:e0126411. 10.1371/journal.pone.0126411 25997015 PMC4440630

[B27] Kennedy H. (1999). *Explosive Secondary Pollen Presentation in Family Marantaceae.* Victoria, BC: Botanical Electronic News 216.

[B28] Kunze H. (1984). Vergleichende Studien an Cannaceen- und Marantaceenblüten. *Flora* 175 301–318. 10.1016/S0367-2530(17)31453-6

[B29] Lindley J. (1819). *Maranta zebrina* stripe-leaved *Maranta*. *Bot. Regist.* 5 385–388. 10.1098/rsta.2016.0183 27354737

[B30] Lindley J. (1826). *Calathea longibracteata* long-bracted *Calathea*. *Bot. Regist.* 12 1020–1021.

[B31] Lockhart J. A. (1967). Physical nature of irreversible deformation of plant cells. *Plant Physiol.* 42 1545–1552. 10.1104/pp.42.11.1545 16656691 PMC1086764

[B32] Nees von Esenbeck C. G. (1831). Über die Gattungen *Maranta* und *Thalia*. *Linnaea* 6 303–342.

[B33] Niklas K. J. (1992). *Plant Biomechanics: An Engineering Approach to Plant Form and Function.* Chicago, IL: The Univesity of Chicago Press.

[B34] Nilsson S. B. Hertz C. H. Falk S. (1958). On the relation between turgor pressure and tissue rigidity. II. *Physiol. Plant.* 11 818–837. 10.1111/j.1399-3054.1958.tb08275.x

[B35] O’Brien T. P. Feder N. McCully M. E. (1964). Polychromatic staining of plant cell walls by toluidine blue O. *Protoplasma* 59 368–373. 10.1007/BF01248568

[B36] Overbeck F. (1924). Studien an den Turgeszenz-Schleudermechanismen von *Dorstenia contrayerva* L. und *Impatiens parviflora* DC. *Jahrbücher Wiss. Bot.* 63:467.

[B37] Petersen O. G. (1890). “Marantaceae,” in *Flora Brasiliensis III* Vol. 3 eds von Martius C. F. P. Eichler A. G. (Munich: R. Oldenbourg), 81–172.

[B38] Pischtschan E. (2007). *Evolutionary Tendencies in Flowers of Marantaceae with Special Reference to the Style Movement Mechanism.* Mainz: University of Mainz.

[B39] Pischtschan E. Claßen-Bockhoff R. (2008). Setting-up tension in the style of Marantaceae. *Plant Biol.* 10 441–450. 10.1111/j.1438-8677.2008.00051.x 18557904

[B40] Pischtschan E. Claßen-Bockhoff R. (2010). Anatomic insights into the thigmonastic style tissue in Marantaceae. *Plant Syst. Evol.* 286 91–102. 10.1007/s00606-010-0282-5

[B41] Pütz N. Froebe H. A. Haese U. (1990). Quantitative Untersuchungen zum Mechanismus der Wurzelkontraktion bei *Acidanthera bicolor* Hochst. (Iridaceae). *Beitr. Biol. Pflanzen* 65 147–161.

[B42] Sapala A. Runions A. Routier-Kierzkowska A. L. Das Gupta M. Hong L. Hofhuis H. (2018). Why plants make puzzle cells, and how their shape emerges. *eLife* 7:e32794. 10.7554/eLife.32794 29482719 PMC5841943

[B43] Skotheim J. M. Mahadevan L. (2005). Physical limits and design principles for plant and fungal movements. *Science* 308 1308–1310. 10.1126/science.1107976 15919993

[B44] Staedler Y. M. Masson D. Schönenberger J. (2013). Plant tissues in 3D via X-ray tomography: simple contrasting methods allow high resolution imaging. *PLoS One* 8:e75295. 10.1371/journal.pone.0075295 24086499 PMC3785515

[B45] Suslov D. Verbelen J. P. (2006). Cellulose orientation determines mechanical anisotropy in onion epidermis cell walls. *J. Exp. Bot.* 57 2183–2192. 10.1093/jxb/erj177 16720609

[B46] Talbot M. White R. (2013). Methanol fixation of plant tissue for scanning electron microscopy improves preservation of tissue morphology and dimensions. *Plant Methods* 9:36. 10.1186/1746-4811-9-36 24083940 PMC3853006

[B47] Umrath K. (1928). Über die Erregungsleitung bei sensitiven Pflanzen, mit Bemerkungen zur Theorie der Erregungsleitung und der elektrischen Erregbarkeit im Allgemeinen. *Planta* 5 274–324. 10.1007/BF01981103

[B48] Weintraub M. (1952). Leaf movements in *Mimosa pudica* L. *New Phytol.* 50 357–382. 10.1111/j.1469-8137.1952.tb05196.x 12223640

[B49] Westermeier A. S. Sachse R. Poppinga S. Vögele P. Adamec L. Speck T. (2018). How the carnivorous waterwheel plant (*Aldrovanda vesiculosa*) snaps. *Proc. R. Soc. B* 285:20180012. 10.1098/rspb.2018.0012 29743251 PMC5966589

