# Supplementary material for: Insights Into the Inside – A Quantitative Histological Study of the Explosively Moving Style in Marantaceae
Source: Front Plant Sci. 2018 Dec 5;9:1695. doi: 10.3389/fpls.2018.01695 (PMC6309734; doi:10.3389/fpls.2018.01695)
Supplement: Supplementary file 1 [file Table_1.pdf]

Supplementary Material

Insights into the inside – a quantitative histological study of the explosively moving style in Marantaceae

Markus Jerominek*, Maria Will, Regine Claßen-Bockhoff

*** Correspondence:** Corresponding Author: info@spinningspecies.com

1 Supplementary Tables

Supplementary Table 1:

Measurements of style length (mm) for the upper and lower side. Mean values and standard deviations are provided for the three different states (U: unreleased, R: released, S: steady), in both fixed and unfixed condition. Relative changes to the unreleased state based on means. As a precondition for the T-test, data were tested for a normal distribution with the Kolmogorov-Smirnov test (K-S-test). Significant differences are indicated by T- or U-tests with P ≤ 0.05 highlighted (bold). Sampling for all measurements: N = 10.E: epidermis; SE: sub-epidermis; se (bold): standard error;

Style side	State (treatment)	Mean	se	Rel. Change to state U	K-S-Test	T-test or U-test
upper side	S (untreated)	5.0	0.3	-0.11	Z=0.198; P=0.200	
	S (FAA)	4.6	0.4	-0.04	Z=0.168; P=0.200	T=2.571; **P=0.019**
	U (untreated)	5.6	0.3		Z=0.233; P=0.133	
	U (FAA)	4.8	0.3		Z=0.140; P=0.200	T=5.175; **P=0.000**
	R (untreated)	4.8	0.2	-0.14	Z=0.176; P=0.200	
	R (FAA)	4.9	0.2	0.02	Z=0.271; P=0.200	T=-0.844; P=0.410
lower side	S (untreated)	7.8	0.3	0.24	Z=0.240; P=0.106	
	S (FAA)	6.7	0.4	0.22	Z=0.194; P=0.200	T=6.888; **P=0.000**
	U (untreated)	6.3	0.3		Z=0.170; P=0.200	
	U (FAA)	5.5	0.3		Z=0.155; P=0.200	T=6.060; **P=0.000**
	R (untreated)	10.7	0.5	0.70	Z=0.335; **P=0.002**	
	R (FAA)	8.8	0.3	0.60	Z=0.271; **P=0.036**	U=-3.780. **P=0.000**

Supplementary Table 2:

Calculation of the correction factors. The reconstructed lengths (mm) of the steady (S). unreleased (U) and released (R) state are calculated by a linear increase from the upper to the lower side. The relative position (Rel. Pos.) of the sector was multiplied with the difference of upper and lower length and added to the length of the upper side **(Difference x Rel. Pos. + Length upper side)**. A correction factor was calculated by the ratio of unfixed and fixed lengths (**Length untreated / Length FAA)**. FAA: Formalin-Acetic-Alcohol.

	Rel. Pos.	Reconstructed length						Correction factor		
State		S	S	U	U	R	R	S	U	R
Treatment		no	FAA	no	FAA	no	FAA			
Upper side	0	5.0	4.6	5.6	4.8	4.8	4.9			
Lower side	1	7.8	6.7	6.3	5.5	10.7	8.8			
Difference		2.8	2.1	0.7	0.7	5.9	3.9			
Epidermis	0	5.0	4.6	5.6	4.8	4.8	4.9	1.09	1.17	0.98
Sub-epidermis	0	5.0	4.6	5.6	4.8	4.8	4.9	1.09	1.17	0.98
Sector 1	0.05	5.1	4.7	5.6	4.8	5.1	5.1	1.09	1.17	1.00
Sector 2	0.15	5.4	4.9	5.7	4.9	5.7	5.5	1.10	1.16	1.04
Sector 3	0.25	5.7	5.1	5.8	5.0	6.3	5.9	1.11	1.16	1.07
Sector 4	0.35	6.0	5.3	5.8	5.0	6.9	6.3	1.12	1.16	1.10
Sector 5	0.45	6.3	5.5	5.9	5.1	7.5	6.7	1.13	1.16	1.12
Sector 6	0.55	6.5	5.8	6.0	5.2	8.0	7.0	1.14	1.15	1.14
Sector 7	0.65	6.8	6.0	6.1	5.3	8.6	7.4	1.14	1.15	1.16
Sector 8	0.75	7.1	6.2	6.1	5.3	9.2	7.8	1.15	1.15	1.18
Sector 9	0.85	7.4	6.4	6.2	5.4	9.8	8.2	1.16	1.15	1.19
Sector 10	0.95	7.7	6.6	6.3	5.5	10.4	8.6	1.16	1.15	1.21

Supplementary Table 3:

C**ell area (µm²) from cross sections.** Relative changes to the unreleased state based on medians. As a precondition for the T-test, data were tested for a normal distribution with the Kolmogorov-Smirnov test (K-S-test). Significant differences between the steady (S), unreleased (U), and released (R) state are provided based on the T- or U-test; P≤0.05 (bold). df: degree of freedom; E: epidermis; SE: sub-epidermis; se (bold): standard error;

Range	State	N	Mean	se	Median	Rel. change to state U	K-S-Test	Sig.	Tested groups	df	T-test or U-Test
E	S	42	157.28	7.39	152.03	0.58	0.085	0.200	S / U	42/85	**U=652; P=0.000**
	U	85	100.55	4.57	96.26		0.132	**0.001**	U / R	85/71	**U=1734.5; P=0.000**
	R	71	129.17	4.41	133.23	0.38	0.092	0.200	R / S	111	**T=-3.487; P=0.001**
SE	S	38	346.61	26.06	311.54	0.42	0.113	0.200	S / U	49.202	**T=4.466; P=0.000**
	U	58	221.10	10.52	218.95		0.092	0.200	U / R	107	**T=-2.050; P=0.043**
	R	51	255.54	13.33	245.69	0.12	0.119	0.070	R / S	56.061	**T=-3.111; P=0.003**
1	S	46	311.34	18.59	306.19	0.00	0.079	0.200	S / U	133	T=-0.365; P=0.716
	U	89	320.59	15.48	304.97		0.093	0.054	U / R	152	T=-0.191; P=0.849
	R	65	324.91	15.87	325.14	0.07	0.074	0.200	R / S	109	T=0.554; P=0.581
2	S	61	302.46	15.96	271.35	-0.32	0.107	0.081	S / U	163	**T=-4.967; P=0.000**
	U	104	416.23	14.83	396.49		0.086	0.057	U / R	61/104	U=4601.5; P=0.477
	R	94	407.91	14.65	396.18	0.00	0.098	**0.026**	R / S	94/61	**U=1647.5; P=0.000**
3	S	66	351.16	17.02	349.28	-0.18	0.075	0.200	S / U	173	**T=-3.798; P=0.000**
	U	109	436.88	14.22	427.81		0.064	0.200	U / R	109/97	U=5020; P=0.533
	R	97	428.56	18.32	423.53	-0.01	0.108	**0.007**	R / S	97/66	**U=2458.5; P=0.012**
4	S	62	386.18	18.47	378.00	-0.02	0.091	0.200	S / U	174	T=-0.508; P=0.612
	U	114	396.69	11.50	385.95		0.049	0.200	U / R	220	T=-1.341; P=0.181
	R	108	421.03	14.15	416.96	0.08	0.056	0.200	R / S	168	T=1.493; P=0.137
5	S	62	329.25	14.04	341.79	0.06	0.082	0.200	S / U	187	T=-0.403; P=0.687
	U	127	336.36	10.24	322.69		0.079	0.053	U / R	217.992	**T=-3.207; P=0.002**
	R	114	390.18	13.30	368.99	0.14	0.078	0.085	R / S	174	**T=2.929; P=0.004**
6	S	62	339.67	15.75	316.89	-0.01	0.088	0.200	S / U	62/127	U=3889.5; P=0.893
	U	127	337.66	10.07	320.55		0.084	**0.029**	U / R	127/120	U=7007; P=0.275
	R	120	326.14	10.79	307.41	-0.04	0.086	**0.031**	R / S	120/62	U=3488; P=0.491
7	S	62	322.69	15.20	317.80	-0.11	0.085	0.200	S / U	184	T=-1.802; P=0.073
	U	124	353.85	9.58	357.83		0.036	0.200	U / R	244	T=0.990; P=0.323
	R	122	339.37	11.07	337.36	-0.06	0.077	0.073	R / S	182	T=0.881; P=0.380
8	S	71	292.63	13.32	280.83	-0.05	0.069	0.200	S / U	205	T=-1.132; P=0.259
	U	136	312.89	10.90	294.88		0.073	0.076	U / R	136/129	U=8728; P=0.944
	R	129	311.20	9.82	297.33	0.01	0.095	**0.006**	R / S	129/71	U=4243; P=0.391
9	S	69	260.26	12.96	238.05	-0.15	0.092	0.200	S / U	206	T=-1.772; P=0.078
	U	139	287.98	8.96	281.13		0.053	0.200	U / R	263	T=-0.659; P=0.511
	R	126	296.55	9.43	288.16	0.02	0.06	0.200	R / S	193	**T=2.276; P=0.024**
10	S	68	209.46	11.42	204.43	-0.01	0.08	0.200	S / U	190	T=-0.421; P=0.674
	U	124	214.89	7.22	206.72		0.063	0.200	U / R	124/105	U=6179; P=0.508
	R	105	221.72	7.43	212.38	0.03	0.087	**0.050**	R / S	105/68	U=3251; P=0.321

Supplementary Table 4:

Corrected cell length (µm) from longitudinal sections. Relative changes to the unreleased state based on medians. As a precondition for the T-test, data were tested for a normal distribution with the Kolmogorov-Smirnov test (K-S-test). Significant differences between the steady (S), unreleased (U), and released (R) state are provided based on the T- or U-test; P≤0.05 (bold); df: degree of freedom; E: epidermis; SE: sub-epidermis; se (bold): standard error;

Range	State	N	Mean	se	Median	Rel. change to state U	K-S-Test	Sig.	Tested groups	df	T-test or U-Test
E	S	62	49.95	1.47	48.51	-0.18	0.077	0.200	S / U	91.913	**T=-3.960; P=0.000**
	U	51	60.16	2.12	59.09		0.113	0.113	U / R	51/66	**U=791; P=0.000**
	R	66	46.68	1.29	44.33	-0.25	0.162	**0.000**	R / S	66/62	U=1653; P=0.061
SE	S	43	67.77	2.34	65.33	-0.20	0.099	0.200	S / U	80	**T=-3.690; P=0.000**
	U	39	82.80	3.41	81.76		0.128	0.106	U / R	75	**T=2.645; P=0.010**
	R	38	71.27	2.69	67.58	-0.17	0.11	0.200	R / S	79	T=0.985; P=0.328
1	S	48	96.25	3.49	95.65	-0.14	0.119	0.085	S / U	102	**T=3.505; P=0.001**
	U	56	113.73	3.52	111.55		0.073	0.200	U / R	110	**T=5.620; P=0.000**
	R	56	88.35	2.84	85.75	-0.23	0.081	0.200	R / S	102	T=-1.775; P=0.079
2	S	46	135.28	7.36	122.84	-0.06	0.139	**0.027**	S / U	46/49	U=988; P=0.301
	U	49	146.28	7.74	131.28		0.132	**0.032**	U / R	49/16	U=361; P=0.637
	R	16	144.69	8.63	148.90	0.13	0.134	0.200	R / S	16/46	U=293; P=0.228
3	S	65	136.43	5.47	130.97	0.22	0.119	**0.022**	S / U	65/104	**U=2264; P=0.000**
	U	104	114.31	3.30	107.01		0.101	**0.011**	U / R	104/71	**U=2451; P=0.000**
	R	71	135.41	4.76	125.60	0.17	0.117	**0.018**	R / S	71/65	U=2280; P=0.905
4	S	150	90.44	1.76	86.08	-0.05	0.088	**0.007**	S / U	176/150	U=12337; P=0.309
	U	176	92.32	1.68	90.59		0.052	0.200	U / R	286	**T=-4.801; P=0.000**
	R	112	106.18	2.47	103.63	0.14	0.076	0.139	R / S	112/150	**U=5353; P=0.000**
5	S	174	88.42	1.61	84.77	-0.01	0.078	**0.012**	S / U	174/190	U=15630; P=0.369
	U	190	86.76	1.61	85.38		0.071	**0.020**	U / R	190/136	**U=8248; P=0.000**
	R	136	105.07	2.51	103.46	0.21	0.079	**0.038**	R / S	136/174	**U=7902; P=0.000**
6	S	192	77.21	1.38	73.84	-0.01	0.078	**0.007**	S / U	192/205	U=19286; P=0.730
	U	205	77.77	1.33	74.93		0.066	**0.030**	U / R	205/136	**U=5245; P=0.000**
	R	136	106.80	2.25	101.91	0.36	0.091	**0.008**	R / S	136/192	**U=4756; P=0.000**
7	S	205	73.14	1.21	70.42	-0.05	0.072	**0.012**	S / U	205/207	U=20006; P=0.316
	U	207	74.82	1.30	73.78		0.058	0.086	U / R	285.803	**T=-11.622; P=0.000**
	R	153	101.27	1.87	98.10	0.33	0.065	0.200	R / S	153/205	**U=5131; P=0.000**
8	S	173	76.77	1.40	73.35	-0.01	0.097	**0.000**	S / U	173/215	U=18589; P=0.994
	U	215	76.32	1.24	73.80		0.068	**0.019**	U / R	215/138	**U=5930; P=0.000**
	R	138	103.36	2.24	99.77	0.35	0.085	**0.015**	R / S	138/173	**U=4918; P=0.000**
9	S	143	80.21	1.46	78.68	0.05	0.071	0.072	S / U	143/179	U=12114; P=0.410
	U	179	78.97	1.41	75.21		0.085	**0.003**	U / R	179/99	**U=3241; P=0.000**
	R	99	111.12	2.99	106.01	0.41	0.088	0.056	R / S	144.577	**T=9.299; P=0.000**
10	S	104	90.88	2.00	87.80	0.07	0.09	**0.039**	S / U	104/115	U=5078; P=0.054
	U	115	85.84	1.96	82.02		0.097	**0.010**	U / R	115/45	**U=711; P=0.000**
	R	45	125.31	4.48	118.31	0.44	0.123	0.084	R / S	45/104	**U=772; P=0.000**

Supplementary Table 5:

Calculation of the volumetric changes. The medians of cell length (µm) and cell area (µm2) were multiplied for each state and range to calculate the corresponding volume. Absolute and relative changes of the steady (S) and released (R) states were given in comparison to the unreleased (U) state.

Range	State	Median cell length in µm	Median cell area in µm^2	Volume in µm^3	absolute Changes to unreleased state	relative Changes to unreleased state
E	S	48.51	152.03	7375	1687	29.6%
	U	59.09	96.26	5688		
	R	44.33	133.23	5906	218	3.8%
SE	S	65.33	311.54	20353	2451	13.7%
	U	81.76	218.95	17902		
	R	67.58	245.69	16602	-1300	-7.3%
1	S	95.65	306.19	29288	-4733	-13.9%
	U	111.55	304.97	34021		
	R	85.75	325.14	27881	-6139	-18.0%
2	S	122.84	271.35	33334	-18716	-36.0%
	U	131.28	396.49	52051		
	R	148.90	396.18	58991	6940	13.3%
3	S	130.97	349.28	45743	-37	-0.1%
	U	107.01	427.81	45780		
	R	125.60	423.53	53194	7414	16.2%
4	S	86.08	378.00	32539	-2423	-6.9%
	U	90.59	385.95	34962		
	R	103.63	416.96	43210	8248	23.6%
5	S	84.77	341.79	28974	1423	5.2%
	U	85.38	322.69	27550		
	R	103.46	368.99	38176	10626	38.6%
6	S	73.84	316.89	23399	-619	-2.6%
	U	74.93	320.55	24019		
	R	101.91	307.41	31328	7309	30.4%
7	S	70.42	317.80	22381	-4019	-15.2%
	U	73.78	357.83	26400		
	R	98.10	337.36	33096	6696	25.4%
8	S	73.35	280.83	20598	-1164	-5.4%
	U	73.80	294.88	21762		
	R	99.77	297.33	29665	7903	36.3%
9	S	78.68	238.05	18729	-2415	-11.4%
	U	75.21	281.13	21144		
	R	106.01	288.16	30548	9404	44.5%
10	S	87.80	204.43	17950	994	5.9%
	U	82.02	206.72	16956		
	R	118.31	212.38	25126	8170	48.2%
2 - 10	U			270624		
	R			343334	72709	26.9%

55

Supplementary Table 6:

Statistics of the vascular bundle length in *G. bachemiana.* Relative changes to the unreleased state based on means. As a precondition for the T-test, data were tested for a normal distribution with the Kolmogorov-Smirnov test (K-S-test). Vascular bundle (VB) length is measured for 10 helical loops (µm). Significant differences between the steady (S), unreleased (U) and released (R) states were calculated with a Mann-Whitney-U-Test; $P \leq 0.05$ (bold); df: degree of freedom; E: epidermis; se: standard error;

	State	N	Mean	se	Rel. Change to state U	K-S-Test	Sig.	Tested groups	df	U-Test
Upper VBs	S	114	45.47	1.04	-0.15	0.145	**0.000**	S / U	114/77	**U=3001; P=0.000**
	U	77	53.72	2.05		0.220	**0.000**	U / R	77/52	U=1668; P=0.109
	R	52	48.29	1.41	-0.10	0.146	**0.007**	R / S	52/114	**U=2279; P=0.017**
Lower VB	S	143	51.86	0.90	-0.07	0.129	**0.000**	S / U	143/150	**U=8849; P=0.010**
	U	150	55.99	1.15		0.186	**0.000**	U / R	150/101	**U=3571; P=0.000**
	R	101	68.46	1.64	0.22	0.119	**0.001**	R / S	101/143	**U=2539; P=0.000**
