# Supplementary material for: Insights Into the Inside – A Quantitative Histological Study of the Explosively Moving Style in Marantaceae
Source: Front Plant Sci. 2018 Dec 5;9:1695. doi: 10.3389/fpls.2018.01695 (PMC6309734; doi:10.3389/fpls.2018.01695)
Supplement: Supplementary file 2 [file Table_2.pdf]

Supplementary Table 2: Calculation of the correction factors. The reconstructed lengths (mm) of the steady (S), unreleased (U) and released (R) state are calculated by a linear increase from the upper to the lower side. The relative position (Rel. Pos.) of the sector was multiplied with the difference of upper and lower length and added to the length of the upper side **(Difference x Rel. Pos. + Length upper side)**. A correction factor was calculated by the ratio of unfixed and fixed lengths (**Length untreated / Length FAA)**. FAA: Formalin-Acetic-Alcohol.

	Rel. Pos.	Reconstructed length						Correction factor		
State		S	S	U	U	R	R	S	U	R
Treatment		no	FAA	no	FAA	no	FAA			
Upper side	0	5.0	4.6	5.6	4.8	4.8	4.9			
Lower side	1	7.8	6.7	6.3	5.5	10.7	8.8			
Difference		2.8	2.1	0.7	0.7	5.9	3.9			
Epidermis	0	5.0	4.6	5.6	4.8	4.8	4.9	1.09	1.17	0.98
Sub-epidermis	0	5.0	4.6	5.6	4.8	4.8	4.9	1.09	1.17	0.98
Sector 1	0.05	5.1	4.7	5.6	4.8	5.1	5.1	1.09	1.17	1.00
Sector 2	0.15	5.4	4.9	5.7	4.9	5.7	5.5	1.10	1.16	1.04
Sector 3	0.25	5.7	5.1	5.8	5.0	6.3	5.9	1.11	1.16	1.07
Sector 4	0.35	6.0	5.3	5.8	5.0	6.9	6.3	1.12	1.16	1.10
Sector 5	0.45	6.3	5.5	5.9	5.1	7.5	6.7	1.13	1.16	1.12
Sector 6	0.55	6.5	5.8	6.0	5.2	8.0	7.0	1.14	1.15	1.14
Sector 7	0.65	6.8	6.0	6.1	5.3	8.6	7.4	1.14	1.15	1.16
Sector 8	0.75	7.1	6.2	6.1	5.3	9.2	7.8	1.15	1.15	1.18
Sector 9	0.85	7.4	6.4	6.2	5.4	9.8	8.2	1.16	1.15	1.19
Sector 10	0.95	7.7	6.6	6.3	5.5	10.4	8.6	1.16	1.15	1.21
