# Supplementary material for: Insights Into the Inside – A Quantitative Histological Study of the Explosively Moving Style in Marantaceae
Source: Front Plant Sci. 2018 Dec 5;9:1695. doi: 10.3389/fpls.2018.01695 (PMC6309734; doi:10.3389/fpls.2018.01695)
Supplement: Supplementary file 3 [file Table_3.pdf]

Supplementary Table 3: Cell area (μm^2) from cross sections. Significant differences between the steady (S), unreleased (U), and released (R) state are provided based on the T- or U-test; P≤0.005 (bold). df: degree of freedom; E: epidermis; SE: sub-epidermis se (bold): standard error.

Sector	State	N	Mean	se	Median	K-S-Test	Sig.	Tested groups	df	T-test or U-Test
E	S	42	157.28	7.39	152.03	0.085	0.200	S / U	42/85	**U = 652; P = 0.000**
	U	85	100.55	4.57	96.26	0.132	**0.001**	U / R	85/71	**U = 1734.5; P = 0.000**
	R	71	129.17	4.41	133.23	0.092	0.200	R / S	111	**T = -3.487; P = 0.001**
SE	S	38	346.61	26.06	311.54	0.113	0.200	S / U	49.202	**T = 4.466; P = 0.000**
	U	58	221.10	10.52	218.95	0.092	0.200	U / R	107	**T = -2.050; P = 0.043**
	R	51	255.54	13.33	245.69	0.119	0.070	R / S	56.061	**T = -3.111; P = 0.003**
1	S	46	311.34	18.59	306.19	0.079	0.200	S / U	133	T = -0.365; P = 0.716
	U	89	320.59	15.48	304.97	0.093	0.054	U / R	152	T = -0.191; P = 0.849
	R	65	324.91	15.87	325.14	0.074	0.200	R / S	109	T = 0.554; P = 0.581
2	S	61	302.46	15.96	271.35	0.107	0.081	S / U	163	**T = -4.967; P = 0.000**
	U	104	416.23	14.83	396.49	0.086	0.057	U / R	61/104	U = 4601.5; P = 0.477
	R	94	407.91	14.65	396.18	0.098	**0.026**	R / S	94/61	**U = 1647.5; P = 0.000**
3	S	66	351.16	17.02	349.28	0.075	0.200	S / U	173	**T = -3.798; P = 0.000**
	U	109	436.88	14.22	427.81	0.064	0.200	U / R	109/97	U = 5020; P = 0.533
	R	97	428.56	18.32	423.53	0.108	**0.007**	R / S	97/66	**U = 2458.5; P = 0.012**
4	S	62	386.18	18.47	378.00	0.091	0.200	S / U	174	T = -0.508; P = 0.612
	U	114	396.69	11.50	385.95	0.049	0.200	U / R	220	T = -1.341; P = 0.181
	R	108	421.03	14.15	416.96	0.056	0.200	R / S	168	T = 1.493; P = 0.137
5	S	62	329.25	14.04	341.79	0.082	0.200	S / U	187	T = -0.403; P = 0.687
	U	127	336.36	10.24	322.69	0.079	0.053	U / R	217.992	**T = -3.207; P = 0.002**
	R	114	390.18	13.30	368.99	0.078	0.085	R / S	174	**T = 2.929; P = 0.004**
6	S	62	339.67	15.75	316.89	0.088	0.200	S / U	62/127	U = 3889.5; P = 0.893
	U	127	337.66	10.07	320.55	0.084	**0.029**	U / R	127/120	U = 7007; P = 0.275
	R	120	326.14	10.79	307.41	0.086	**0.031**	R / S	120/62	U = 3488; P = 0.491
7	S	62	322.69	15.20	317.80	0.085	0.200	S / U	184	T = -1.802; P = 0.073
	U	124	353.85	9.58	357.83	0.036	0.200	U / R	244	T = 0.990; P = 0.323
	R	122	339.37	11.07	337.36	0.077	0.073	R / S	182	T = 0.881; P = 0.380
8	S	71	292.63	13.32	280.83	0.069	0.200	S / U	205	T = -1.132; P = 0.259
	U	136	312.89	10.90	294.88	0.073	0.076	U / R	136/129	U = 8728; P = 0.944
	R	129	311.20	9.82	297.33	0.095	**0.006**	R / S	129/71	U = 4243; P = 0.391
9	S	69	260.26	12.96	238.05	0.092	0.200	S / U	206	T = -1.772; P = 0.078
	U	139	287.98	8.96	281.13	0.053	0.200	U / R	263	T = -0.659; P = 0.511
	R	126	296.55	9.43	288.16	0.06	0.200	R / S	193	**T = 2.276; P = 0.024**
10	S	68	209.46	11.42	204.43	0.08	0.200	S / U	190	T = -0.421; P = 0.674
	U	124	214.89	7.22	206.72	0.063	0.200	U / R	124/105	U = 6179; P = 0.508
	R	105	221.72	7.43	212.38	0.087	**0.050**	R / S	105/68	U = 3251; P = 0.321
