# Supplementary material for: Insights Into the Inside – A Quantitative Histological Study of the Explosively Moving Style in Marantaceae
Source: Front Plant Sci. 2018 Dec 5;9:1695. doi: 10.3389/fpls.2018.01695 (PMC6309734; doi:10.3389/fpls.2018.01695)
Supplement: Supplementary file 4 [file Table_4.pdf]

Supplementary Table 4: Corrected cell length (µm) from longitudinal sections. Significant differences between the steady (S), unreleased (U), and released (R) state are provided based on the T- or U-test; P≤0.005 (bold); abbreviations according to Tab. S3.

Sector	State	N	Mean	se	Median	K-S-Test	Sig.	Tested groups	df	T-test or U-Test
E	S	62	49.95	1.47	48.51	0.077	0.200	S / U	91.913	**T = -3.960; P = 0.000**
	U	51	60.16	2.12	59.09	0.113	0.113	U / R	51/66	**U = 791; P = 0.000**
	R	66	46.68	1.29	44.33	0.162	**0.000**	R / S	66/62	U = 1653; P = 0.061
SE	S	43	67.77	2.34	65.33	0.099	0.200	S / U	80	**T = -3.690; P = 0.000**
	U	39	82.80	3.41	81.76	0.128	0.106	U / R	75	**T = 2.645; P = 0.010**
	R	38	71.27	2.69	67.58	0.11	0.200	R / S	79	T = 0.985; P = 0.328
1	S	48	96.25	3.49	95.65	0.119	0.085	S / U	102	**T = 3.505; P = 0.001**
	U	56	113.73	3.52	111.55	0.073	0.200	U / R	110	**T = 5.620; P = 0.000**
	R	56	88.35	2.84	85.75	0.081	0.200	R / S	102	T = -1.775; P = 0.079
2	S	46	135.28	7.36	122.84	0.139	**0.027**	S / U	46/49	U = 988; P = 0.301
	U	49	146.28	7.74	131.28	0.132	**0.032**	U / R	49/16	U = 361; P = 0.637
	R	16	144.69	8.63	148.90	0.134	0.200	R / S	16/46	U = 293; P = 0.228
3	S	65	136.43	5.47	130.97	0.119	**0.022**	S / U	65/104	**U = 2264; P = 0.000**
	U	104	114.31	3.30	107.01	0.101	**0.011**	U / R	104/71	**U = 2451; P = 0.000**
	R	71	135.41	4.76	125.60	0.117	**0.018**	R / S	71/65	U = 2280; P = 0.905
4	S	150	90.44	1.76	86.08	0.088	**0.007**	S / U	176/150	U = 12337; P = 0.309
	U	176	92.32	1.68	90.59	0.052	0.200	U / R	286	**T = -4.801; P = 0.000**
	R	112	106.18	2.47	103.63	0.076	0.139	R / S	112/150	**U = 5353; P = 0.000**
5	S	174	88.42	1.61	84.77	0.078	**0.012**	S / U	174/190	U = 15630; P = 0.369
	U	190	86.76	1.61	85.38	0.071	**0.020**	U / R	190/136	**U = 8248; P = 0.000**
	R	136	105.07	2.51	103.46	0.079	**0.038**	R / S	136/174	**U = 7902; P = 0.000**
6	S	192	77.21	1.38	73.84	0.078	**0.007**	S / U	192/205	U = 19286; P = 0.730
	U	205	77.77	1.33	74.93	0.066	**0.030**	U / R	205/136	**U = 5245; P = 0.000**
	R	136	106.80	2.25	101.91	0.091	**0.008**	R / S	136/192	**U = 4756; P = 0.000**
7	S	205	73.14	1.21	70.42	0.072	**0.012**	S / U	205/207	U = 20006; P = 0.316
	U	207	74.82	1.30	73.78	0.058	0.086	U / R	285.803	**T = -11.622; P = 0.000**
	R	153	101.27	1.87	98.10	0.065	0.200	R / S	153/205	**U = 5131; P = 0.000**
8	S	173	76.77	1.40	73.35	0.097	**0.000**	S / U	173/215	U = 18589; P = 0.994
	U	215	76.32	1.24	73.80	0.068	**0.019**	U / R	215/138	**U = 5930; P = 0.000**
	R	138	103.36	2.24	99.77	0.085	**0.015**	R / S	138/173	**U = 4918; P = 0.000**
9	S	143	80.21	1.46	78.68	0.071	0.072	S / U	143/179	U = 12114; P = 0.410
	U	179	78.97	1.41	75.21	0.085	**0.003**	U / R	179/99	**U = 3241; P = 0.000**
	R	99	111.12	2.99	106.01	0.088	0.056	R / S	144.577	**T = 9.299; P = 0.000**
10	S	104	90.88	2.00	87.80	0.09	**0.039**	S / U	104/115	U = 5078; P = 0.054
	U	115	85.84	1.96	82.02	0.097	**0.010**	U / R	115/45	**U = 711; P = 0.000**
	R	45	125.31	4.48	118.31	0.123	0.084	R / S	45/104	**U = 772; P = 0.000**
