# Supplementary material for: Insights Into the Inside – A Quantitative Histological Study of the Explosively Moving Style in Marantaceae
Source: Front Plant Sci. 2018 Dec 5;9:1695. doi: 10.3389/fpls.2018.01695 (PMC6309734; doi:10.3389/fpls.2018.01695)
Supplement: Supplementary file 5 [file Table_5.pdf]

Supplementary Table 5: Calculation of the volumetric changes. The medians of cell length (µm) and cell area (µm2) were multiplied for each state and range to calculate the corresponding volume. Absolute and relative changes of the steady (S) and released (R) states were given in comparison to the unreleased (U) state.

Range	State	Median cell length in µm	Median cell area in µm^2	Volume in µm^3	absolute Changes to unreleased state	relative Changes to unreleased state
E	S	48.51	152.03	7375	1687	29.6%
	U	59.09	96.26	5688		
	R	44.33	133.23	5906	218	3.8%
SE	S	65.33	311.54	20353	2451	13.7%
	U	81.76	218.95	17902		
	R	67.58	245.69	16602	-1300	-7.3%
1	S	95.65	306.19	29288	-4733	-13.9%
	U	111.55	304.97	34021		
	R	85.75	325.14	27881	-6139	-18.0%
2	S	122.84	271.35	33334	-18716	-36.0%
	U	131.28	396.49	52051		
	R	148.90	396.18	58991	6940	13.3%
3	S	130.97	349.28	45743	-37	-0.1%
	U	107.01	427.81	45780		
	R	125.60	423.53	53194	7414	16.2%
4	S	86.08	378.00	32539	-2423	-6.9%
	U	90.59	385.95	34962		
	R	103.63	416.96	43210	8248	23.6%
5	S	84.77	341.79	28974	1423	5.2%
	U	85.38	322.69	27550		
	R	103.46	368.99	38176	10626	38.6%
6	S	73.84	316.89	23399	-619	-2.6%
	U	74.93	320.55	24019		
	R	101.91	307.41	31328	7309	30.4%
7	S	70.42	317.80	22381	-4019	-15.2%
	U	73.78	357.83	26400		
	R	98.10	337.36	33096	6696	25.4%
8	S	73.35	280.83	20598	-1164	-5.4%
	U	73.80	294.88	21762		
	R	99.77	297.33	29665	7903	36.3%
9	S	78.68	238.05	18729	-2415	-11.4%
	U	75.21	281.13	21144		
	R	106.01	288.16	30548	9404	44.5%
10	S	87.80	204.43	17950	994	5.9%
	U	82.02	206.72	16956		
	R	118.31	212.38	25126	8170	48.2%
2 - 10	U			270624		
	R			343334	72709	26.9%
