# Supplementary material for: Insights Into the Inside – A Quantitative Histological Study of the Explosively Moving Style in Marantaceae
Source: Front Plant Sci. 2018 Dec 5;9:1695. doi: 10.3389/fpls.2018.01695 (PMC6309734; doi:10.3389/fpls.2018.01695)
Supplement: Supplementary file 6 [file Table_6.pdf]

Supplementary Table 6: Statistics of the vascular bundle length in *G. bachemiana.* Vascular bundle (VB) length is measured for 10 helical loops (µm). Significant differences between the steady (S), unreleased (U), and released (R) states were calculated with a Mann-Whitney-U-Test; P ≤ 0.005 (bold); abbreviations according to Tab. S3.

	State	N	Mean	se	K-S-Test	Sig.	Tested groups	df	U-Test
Upper VBs	S	114	45.47	1.04	0.145	**0.000**	S / U	114/77	**U = 3001; P = 0.000**
	U	77	53.72	2.05	0.220	**0.000**	U / R	77/52	U = 1668; P = 0.109
	R	52	48.29	1.41	0.146	**0.007**	R / S	52/114	**U = 2279; P = 0.017**
Lower VB	S	143	51.86	0.90	0.129	**0.000**	S / U	143/150	**U = 8849; P = 0.010**
	U	150	55.99	1.15	0.186	**0.000**	U / R	150/101	**U = 3571; P = 0.000**
	R	101	68.46	1.64	0.119	**0.001**	R / S	101/143	**U = 2539; P = 0.000**
